# Mitochondrial genomes and genetic structure of the Kemp's ridley sea turtle (*Lepidochelys kempii*)

**DOI:** 10.1002/ece3.5891

**Published:** 2019-12-05

**Authors:** Hilary R. Frandsen, Diego F. Figueroa, Jeff A. George

**Affiliations:** ^1^ University of Texas Rio Grande Valley Brownsville Texas; ^2^ Sea Turtle, Inc. South Padre Island Texas

**Keywords:** conservation, control region, genetic diversity, next generation sequencing, phylogeny, phylogeography

## Abstract

The Kemp's ridley (*Lepidochelys kempii*) is the world's most endangered sea turtle species and is primarily distributed in the Gulf of Mexico. In the United States, South Padre Island, Texas serves as a key nesting ground for the species. Genetic studies of the Kemp's ridley have been used to aid in conservation and management practices, with the mitochondrial control region as the most commonly used marker due to its perceived hypervariability and ease of sequencing. However, with the advent of next generation sequencing technology, targeting complete mitochondrial genomes is now feasible. Here, we describe a more complete mitochondrial genome for the Kemp's ridley than has been previously published in literature and demonstrate a cost‐effective and efficient method for obtaining complete mitochondrial genomes from sea turtles. We compare the genetic diversity and taxonomic resolution obtained from whole mitochondrial genomes to that obtained from the mitochondrial control region alone. We compare current genetic diversity with previous records. Furthermore, we evaluate the genetic structure between the breeding stock in South Padre Island and that of deceased Kemp's ridleys recovered on the Northern coast of the Gulf of Mexico after the 2010 BP *Deepwater Horizon* oil spill, and of Kemp's ridleys stranded on the East Coast of the United States. Our results show that complete mitochondrial genomes provide greater resolution than the control region alone. They also show that the genetic diversity of the Kemp's ridley has remained stable, despite large population declines, and that the genetic makeup of deceased turtles stranded after the *Deepwater Horizon* oil spill is indistinguishable from the breeding stock in South Padre Island, Texas.

**Open Data Badge:**

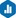

This article has earned an Open Data Badge for making publicly available the digitally‐shareable data necessary to reproduce the reported results. The data is available at https://www.ncbi.nlm.nih.gov/genbank/.

## Graphical abstract

The genetic diversity of Kemp's ridley sea turtle (*Lepidochelys kempii*) in the northwestern Gulf of Mexico is relatively stable despite recent and severe population decline, and the genetic makeup of deceased turtles from the Deepwater Horizon oil spill is virtually identical to that of the breeding stock in South Padre Island, TX. Using the mitochondrial control region to study genetic patterns of this sea turtle is necessary to compare the data to older records, but it should be replaced by targeting complete mitochondrial genomes. The complete mitochondrial region results in more genetic resolution while still providing the sequence for the control region for historical comparisons.

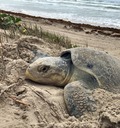

## 1 INTRODUCTION

The Kemp's ridley (*Lepidochelys kempii*), the world's most endangered sea turtle species (Burchfield, 2005), is restricted primarily to the Gulf of Mexico (GoM) and parts of the Northern Atlantic (Bowen et al., 1998). By the mid‐1980s, the Kemp's ridley had suffered a dramatic population decline, and it is estimated that fewer than 300 females nested in 1985 (National Marine Fisheries Service [NMFS] et al., 2011). The species recovered through a combination of domestic and international actions including establishing a bi‐national working agreement between the U.S. and Mexico to increase the protection of nesting females, hatchlings, and eggs (Márquez Millan, Olmeda, Sánchez, & Díaz, 1989; Woody, 1989), prohibition of trawling in GoM waters offshore of Rancho Nuevo, the primary nesting beach in Mexico, during the nesting season (Márquez Millan et al., 1989), a reintroduction program, with a head‐starting component, designed to form a secondary nesting colony at Padre Island National Seashore, Texas (Caillouet, Shaver, & Landry, 2015; Fletcher, 1989; Shaver & Caillouet, 2015), and gradual implementation of turtle excluder devices (TEDS) on U.S. shrimping vessels in the GoM (Turtle Expert Working Group [TEWG], 2000). Positive population growth was observed from the 1980s until 2010 (Caillouet, 2011; Crowder & Heppell, 2011; Gallaway et al., 2013), when annual nesting numbers dropped by 35.4% (Caillouet, Gallaway, & Putman, 2016). A decline in nesting numbers was evident in 2013 and 2014 (Caillouet, 2014; Caillouet et al., 2015), and the overall population was predicted to be decreasing by 5% per year (Heppell, 2014). The causes of the post‐2010 nesting setback are still debated (Caillouet et al., 2018), but recently a record high number of nests were documented in Mexico and Texas in 2017 (Caillouet et al., 2018).

One way to predict the ability of a species to adapt to environmental change is to quantify the genetic variability within the population (Frankham, 1996). Variation within sea turtle DNA is commonly studied using microsatellite markers (Aggarwal et al., 2004), nuclear markers (Bowen et al., 1998; Bowen, Meylan, & Avise, 1991), single nucleotide polymorphisms (Hurtado et al., 2016), or the mitochondrial control region (Gaos et al., 2016; Matsuzawa et al., 2016). Previous studies of Kemp's ridleys have utilized various nuclear markers to determine divergence from Olive ridley (*Lepidochelys olivacea*) sea turtles (Bowen et al., 1998, 1991), determine genetic diversity between nesting colonies (Kichler, 1996), document nesting (Johnson, Bass, Libert, Marshall, & Fulk, 1999), and detect multiple paternity in clutches (Kichler, Holder, Davis, Márquez‐M, & Owens, 1999). Through analysis of heterozygosity at microsatellite loci, the original decline of the Kemp's ridley population was determined to not have had a significant effect on their genetic diversity by Kichler (1996). However, a later study conducted by Stephens (2003) using microsatellites indicated that the demographic bottleneck led to a measurable loss of genetic variation in the species. The apparent contradictions are potentially resolved if the bottleneck occurred too quickly to be detected by Kichler's (1996) markers.

Dutton, Pease, and Shaver (2006) used mitochondrial DNA control region sequences to compare haplotype frequencies of nesting females in Texas to haplotype frequencies from females at Rancho Nuevo, Mexico. The study found six distinct haplotypes; however, the results indicated genetic homogeneity between the two populations. Studies after the 2010 halt in population growth have focused on determining genetic diversity between nesting colonies (Rivera, 2012) and distinguishing individual nesters (Frey, Dutton, Shaver, Shelby Walker, & Rubio, 2014). Microsatellites showed no genotype segregation among rookeries in Tamaulipas, Mexico (Rivera, 2012). Recent work using mitochondrial DNA concluded that there are at least two lineages of females nesting along the Texas coast and discovered eight haplotype sequences for Kemp's ridleys (Frey et al., 2014). Presently, only two partial mitochondrial genomes have been published for Kemp's ridley sea turtles, neither of which could sequence a distinct 117 bp region (Duchene et al., 2012).

Despite the many discrete haplotypes discovered in past studies, the samples taken from Kemp's ridleys in Texas and Mexico indicate there is one homogenous population in the GoM, and though there is infrequent nesting in other areas (Johnson et al., 1999; Marquez‐M, 1994; Rafferty, Shaver, Frandsen, & Montello, 2019), individuals nesting outside of the historic nesting range likely originate from nesting beaches in the western Gulf. Following the dramatic decrease in nesting numbers in 2010, and lowered nesting averages in 2013–2015, evaluation of the genetic diversity of the individuals within the population is highly relevant to investigate the plausibility of a genetic bottleneck and determine the reproductive stability of the Kemp's ridley sea turtle. The decrease in nesting numbers in 2010 may have had an effect on the genetic diversity within the species, but past studies are conflicting (Kichler, 1996; Stephens, 2003).

One method that can be used to determine whether there has been a bottleneck in the Kemp's ridley population is to determine which haplotypes are present within current individuals by analyzing the mitochondrial DNA, and then comparing observed haplotype frequencies to historical data. The control region is thought to be the most variable region within the mitochondrial genome, and targeting this region by using Sanger sequencing has traditionally been more cost effective than sequencing full mitochondrial genomes. However, with the advent of sequencing‐by‐synthesis, now commonly applied in what is known as next generation sequencing, the financial advantage of targeting short markers is quickly diminishing., In sea turtles, complete mitochondrial genomes have been recovered primarily by targeting overlapping fragments of the mitochondrial genome using various sets of primers and sequencing products by the Sanger method (i.e., Drosopoulou et al., 2012; Hernández‐Fernández, Beltrán‐Torres, & Mariño‐Ramírez, 2017; Shamblin et al., 2012), and by using long range PCR and amplifying a few long, overlapping, regions followed by next generation sequencing (i.e., Duchene et al., 2012). The need for amplification through PCR can be bypassed, and genomic DNA extractions can be sequenced directly by using sequencing‐by‐synthesis with the complete mitochondrial genome recovered through assembly of the resulting reads. This method is possible due to the high copy number of mitochondrial DNA contained within genomic DNA, and it has been applied successfully in a wide range of taxa including invertebrates, birds, mammals, amphibians, and reptiles (Cao, Wang, Ge, & Gong, 2019; Caparroz et al., 2018; Chen, 2018; Cho et al., 2018; Cooke, King, Johnson, Boles, & Major, 2012; Figueroa & Baco, 2014; Gao et al., 2017; Huang et al., 2014). We follow a similar procedure as these studies by preparing DNA libraries from genomic DNA extractions followed by next generation sequencing to obtain complete mitochondrial genomes of *L. kempii*.

Here, we describe the complete mitochondrial genomes for several individuals of Kemp's ridley and demonstrate a cost‐effective and efficient method for obtaining complete mitochondrial genomes from sea turtles using next generation sequencing technology. We compare the genetic diversity and taxonomic resolution obtained from whole mitochondrial genomes to that obtained from the mitochondrial control region alone, by evaluating a sampling of Kemp's ridleys in South Padre Island, Texas. Furthermore, using the control region, we evaluate the genetic structure between the breeding stock in South Padre Island and that of deceased Kemp's ridleys recovered on the Northern GoM coast after the 2010 BP *Deepwater Horizon* (DWH) oil spill and of stranded Kemp's ridleys recovered on the East Coast of the United States.

## 2 MATERIALS AND METHODS

### 2.1 Tissue collection

**Figure 1:**
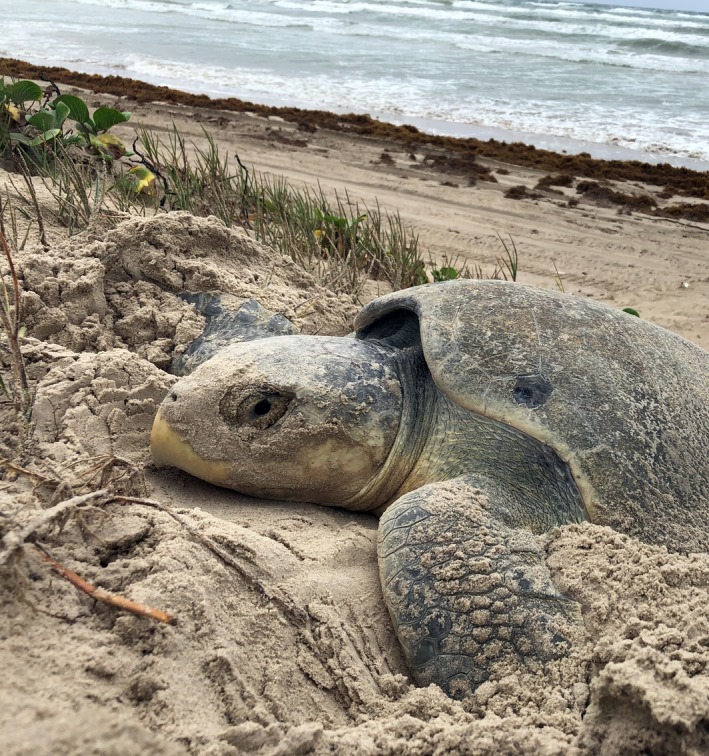
Kemp's ridley (*Lepidochelys kempii*) sea turtle nesting on the south Texas coast. Photographed by Hilary R. Frandsen

**Figure 2:**
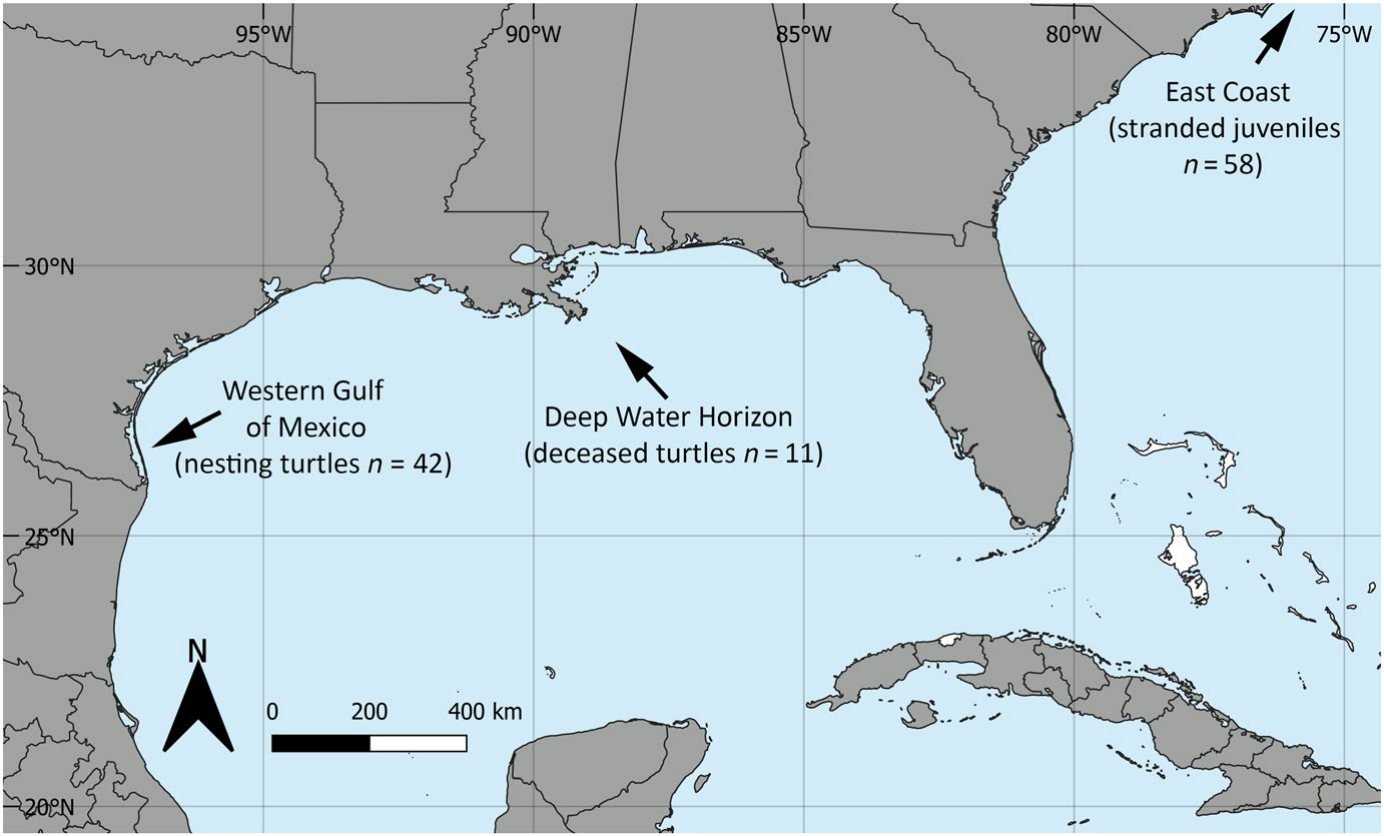
Sources of *Lepidochelys kempii* samples: Nesting females from South Padre Island in the Western Gulf of Mexico, Texas; deceased turtles recovered from the Deep Water Horizon oil spill; and stranded juvenile turtles recovered from the East Coast of the United States

From 2015 to 2016, opportunistic DNA tissue samples were collected from nesting and stranded Kemp's ridleys (Figures 1 and 2) on South Padre Island and Boca Chica Beach, TX. Tissue samples were collected from rehabilitating juvenile Kemp's ridleys at the Georgia Sea Turtle Center (GTSC) on Jekyll Island, GA. These turtles were initially stranded off the coast of Massachusetts during a cold‐stun event. Tissue samples were donated from one captive Kemp's ridley at Sea Turtle Inc. in South Padre Island, TX, and one at Jenkinson's Aquarium in Point Pleasant Beach, NJ. The New England Aquarium (NEAQ) in Boston, MA, donated tissue samples from necropsied cold‐stunned Kemp's ridleys stranded on the coast of Massachusetts. Additionally, tissue samples collected after the 2010 BP DWH oil spill in the GoM were donated from the National Oceanic and Atmospheric Administration's (NOAA) National Marine Fisheries Service (NMFS). These samples were collected from stranded Kemp's ridleys primarily in the Northern GoM, on Alabama, Florida, Louisiana, Mississippi, and Texas coasts.

Tissue samples were obtained by biopsying the rear right flipper using a sterile biopsy one punch, following established protocols (National Oceanic and Atmospheric Administration (NOAA) 2015). To prevent infection, the sampling site was cleaned before and after sampling with povidone‐iodine and alcohol swabsticks. Tissue samples collected from nesting Kemp's ridleys were placed into plastic cryovials containing 95% ethanol and were kept chilled until transfer to a −20°C freezer. Samples collected from juvenile Kemp's ridleys at the GSTC were placed into plastic vials containing saturated NaCl with 20% DMSO and were shipped within 24 hr. The samples were transferred from the saturated NaCl with 20% DMSO to 95% ethanol when received and were stored at −20°C. Samples collected after the DWH oil spill were donated in their original collection containers, including I‐Chem jars and aluminum foil within Ziploc bags. Subsamples were taken from the donated muscle tissue using sterilized blades and were immediately utilized for DNA extraction. Tissue samples donated from NEAQ arrived as flipper clippings stored individually in small Ziploc bags. Subsampling was conducted using sterilized blades, and subsamples were immediately utilized for DNA extraction.

The collected samples were assigned to two geographic areas according to the sampling location (Figure 2): U.S. East Coast (*n* = 58) and Western GoM (*n* = 42). Samples collected on the Northern GoM coast (*n* = 11) were labeled as DWH samples due to uncertainty whether the sampled individuals were transients or residents of the area where they stranded. Samples collected from Kemp's ridleys in rehabilitation facilities (*n* = 2) were labeled as captive, due to their nonreleasable status (Figure 2).

### 2.2 DNA extraction

DNA (200 ng) was obtained from all samples after standard extraction with Thermo Fisher Scientific's Purelink Genomic DNA extraction kit (model #K1820‐01, Thermo Fisher Scientific), following the manufacture's protocol for mouse tissue. Samples were fully digested before extraction for 2–8 hr with Proteinase K. Once the DNA was obtained using the Genomic DNA extraction kit, the concentration of DNA was measured using a Life Technologies Qubit fluorometer (Life Technologies Inc). Gel electrophoresis in a 2% agarose gel stained with ethidium bromide ensured the extracted DNA of all samples was of high quality and high molecular weight. Extracted DNA was stored at −20°C.

### 2.3 Control region

Two sea turtle‐specific primers for the control region sequence were used as follows: LCM15382 (5′ GCTTAACCCTAAAGCATTGG 3′) and H950g (5′ GTCTCGGATTTAGGGGTTTG 3′) (Abreu‐Grobois et al., 2006; LeRoux et al., 2012). A 25 µl PCR reaction containing 17.4 µl of PCR water, 2.5 µl of 10X *Taq* Reaction Buffer, 2.0 µl of 10 mM dNTPs, 1.0 µl of 10 µM Forward Primer, 1.0 µl of 10 µM Reverse Primer, 0.125 µl of Dream*Taq* DNA Polymerase, and 1.0 µl of DNA was run on an Eppendorf Mastercycler pro thermocycler. The following parameters were used: (a) 2 min of initial denaturation at 94°C, (b) 50 s of DNA denaturation at 94°C for 36 cycles, (c) 2 min of primer annealing at 52°C, (d) 90 s of primer extension at 72°C, and (e) 5 min of primer extension at 72°C (Dutton et al., 2008). Gel electrophoresis was used to verify target size and single‐band amplification. PCR products were purified using Sigma‐Aldrich's GenElute PCR Clean‐Up kit or Invitrogen's Purelink Quick PCR Purification Kit. Each extracted PCR product was sequenced in the forward and reverse direction using the LCM15382 (forward) and H950g (reverse) primers by Eurofins MWG Operon, LLC. A consensus sequence for the control region was created using the LCM15382 (forward) and H950g (reverse) primers with Qiagen CLC Genomics Workbench software.

During the alignment of the forward and reverse sequences for each sample, a manual check was conducted to ensure the quality of the chromatogram reading of nucleotides. When there was a conflict between forward and reverse sequences, the strand with the clearest chromatogram trace was given priority, and that nucleotide was assigned as the consensus nucleotide. For those samples that only had one readable strand, that reading was used as the consensus sequence, as long as the chromatogram trace was of excellent quality (no double peaks) and with a minimum Phred score of 20.

### 2.4 Mitochondrial genome

The genomic DNA extraction of ten individuals was used to prepare an indexed library following standard procedures with the Nextera X2 kit. These 10 libraries, along with 86 libraries from other projects, were multiplexed and sequenced on a 100 bp paired‐end lane of Illumina HiSeq 2500 at Harvard's Biopolymers facility. The sequences were de‐multiplexed according to their indices. De novo assemblies were conducted using the software CLC Genomics Workbench. Default settings were used with reads mapped back to contigs (mismatch cost = 2, insertion cost = 3, deletion cost = 3, length fraction = 0.5, similarity fraction = 0.8). The sequences obtained from the assemblies included the full mitochondrial genome for each specimen with an average read coverage of over 100 and a minimum coverage of 35. The assembled genomes were annotated using Qiagen CLC Genomics Workbench software, referencing the two previously published partial Kemp's ridley genomes on GenBank (JX454981 and JX454982).

### 2.5 Phylogenetic and population analyses

Two separate datasets were analyzed, one only using complete mitochondrial genomes and the other only using the mitochondrial control region. All available sequences from GenBank were added to these two datasets (Table 1). Haplotypes were defined with DnaSP software (Rozas, 2009). Minimum‐spanning haplotype networks were created using Population Analysis with Reticulate Trees (PopArt) software (Leigh & Bryant, 2015). Arlequin v3.5.1.2 (Excoffier & Lischer, 2010) was used to make pairwise fixation index (ΦST) comparisons among all sampling groups using default settings. The statistical significance of the fixation indices was assessed under the null hypothesis of panmixia by performing 10,000 permutations of the original dataset by random reallocation of individuals to each population.

**Table 1:** Sequence IDs, sequenced regions, and GenBank accession numbers of *Lepidochelys kempii* and *Lepidochelys olivacea* samples used in study

Sequence ID	Sequenced region	Citation	GenBank accession number
Haplotype 1	Control region	This Study	MN159143
Haplotype 2	Control region	This Study	MN159144
Haplotype 3	Control region	This Study	MN159145
Haplotype 4	Control region	This Study	MN159146
Haplotype 5	Control region	This Study	MN159147
Haplotype 6	Control region	This Study	MN159148
Haplotype 7	Control region	This Study	MN159149
Haplotype 8	Control region	This Study	MN159150
Haplotype 9	Control region	This Study	MN159151
Haplotype 10	Control region	This Study	MN159152
LK 1.1	Control region	Frey et al. (2014)	KF385935
LK 2.1	Control region	Frey et al. (2014)	KF385936
LK 3.1	Control region	Frey et al. (2014)	KF385937
LK 4.1	Control region	Frey et al. (2014)	KF385938
LK 5.1	Control region	Frey et al. (2014)	KF385939
LK 6.1	Control region	Frey et al. (2014)	KF385940
LK 6.2	Control region	Frey et al. (2014)	KF385941
LK 7.1	Control region	Frey et al. (2014)	KF385942
SPI Nest 1	Full mitochondrial genome	This Study	MN136055
SPI Nest 3	Full mitochondrial genome	This Study	MN136058
SPI Nest 6	Full mitochondrial genome	This Study	MN136060
SPI Nest 13	Full mitochondrial genome	This Study	MN136059
SPI Nest 14	Full mitochondrial genome	This Study	MN136053
SPI Nest 16	Full mitochondrial genome	This Study	MN136052
SPI Nest 23	Full mitochondrial genome	This Study	MN136061
SPI Nest 27	Full mitochondrial genome	This Study	MN136056
East Coast BR1	Full mitochondrial genome	This Study	MN136054
East Coast MT1	Full mitochondrial genome	This Study	MN136057
*L. kempii* isolate 68,090	Partial mitochondrial genome	Duchene et al. (2012)	JX454981
*L. kempii* isolate 68,091	Partial mitochondrial genome	Duchene et al. (2012)	JX454982
*L. olivacea* 1	Full mitochondrial genome	Tandon, Trivedi, and Kashyap (2006)	AM258984
*L. olivacea* 2	Full mitochondrial genome	Tandon et al. (2006)	DQ486893

Phylogenetic analyses of the haplotypes identified from the control region were performed with MEGA7 (Kumar, Stecher, & Tamura, 2016) using maximum‐likelihood (ML) methods with bootstrap values from 10,000 replicates. The Tamura 3‐parameter model (Tamura, 1992) with uniform rates was selected by MEGA7 as the best fitting model of molecular evolution based on the Akaike information criterion (AIC). The tree was rooted using two Olive ridley genomes: GenBank accession numbers AM258984 and DQ486893.

The ten mitochondrial genomes were used in a partitioned maximum‐likelihood phylogenetic analysis using PartitionFinder v1.1.1 (Lanfear, Calcott, Kainer, Mayer, & Stamatakis, 2014) and RAxML v8.0.0 (Stamatakis, 2014). Each gene, RNA, and control region were aligned separately using MUSCLE. The resulting alignments were then concatenated. Data blocks were defined by codon positions of the 12 protein‐coding genes, the 2 RNAs, and the control region (Table 2). PartitionFinder divided the data into 8 partitions and selected General Time Reversible plus Gamma (GTR + G) as the best evolutionary model (Table 3). Within the RAxML program, 20 independent searches of 10,000 bootstrap replicates delivered the best maximum‐likelihood (ML) tree. The tree was rooted using two Olive ridley genomes: GenBank accession numbers AM258984 and DQ486893.

**Table 2:** Data block arrangement for partitioned phylogenetic analyses of 12 protein‐coding genes, 2 RNAs, and control region in the *Lepidochelys kempii* mitochondrial genome

Region	Codon positions		
	1	2	3
ND1	2,602–3,574	2,603–3,574	2,604–3,574
ND2	3,575–4,616	3,576–4,616	3,577–4,616
Cox1	4,617–6,169	4,618–6,169	4,619–6,169
Cox2	6,170–6,862	6,171–6,862	6,172–6,862
Atp8	6,863–7,048	6,864–7,048	6,865–7,048
Atp6	7,049–7,734	7,050–7,734	7,051–7,734
Cox3	7,735–8,521	7,736–8,521	7,737–8,521
ND3	8,522–8,871	8,523–8,871	8,524–8,871
ND4L	8,872–9,172	8,873–9,172	8,874–9,172
ND4	9,173–10,553	9,174–10,553	9,175–10,553
ND5	10,554–12,361	10,555–12,361	10,556–12,361
Cytb	12,362–13,507	12,363–13,507	12,364–13,507
ND6	13,508–14,033	13,509–14,033	13,510–14,033
Noncoding			
12s	1–975		
16s	976–2,601		
Control	14,034–14,813		

**Table 3:** Partition scheme identified by PartitionFinder for *Lepidochelys kempii* mitochondrial genome data

Subset	Best model	Subset partitions	Subset sites
1	GTR + G	12s, 16s, ND2^1^	1–975; 976–2601; 3575–4616
2	GTR + G	Cox2^3^, Cox3^1^, Cytb^2^, ND1^1^, ND4L^1^, ND5^2^	2602–3574; 6172–6862; 7735–8521; 8872–9172; 10555–12361; 12363–13507
3	GTR + G	Cox1^1^, ND1^2^, ND3^2^, ND4^1^, ND4L^2^, ND5^3^	2603–3574; 4617–6169; 8523–8871; 8873–9172; 9173–10553; 10556–12361
4	GTR + G	Atp8^2^, Atp8^3^, Cox1^3^, Cox2^2^, Cox3^3^, Cytb^1^, ND1^3^, ND2^2^, ND3^1^, ND3^3^, ND4^2^, ND4L^3^, ND5^1^	2604–3574; 3576–4616; 4619–6169; 6171–6862; 6864–7048; 6865–7048; 7737–8521; 8522–8871; 8524–8871; 8874–9172; 9174–10553; 10554–12361; 12362–13507
5	GTR + G	Atp8^1^, ND2^3^, ND4^3^	3577–4616, 6863–7048, 9175–10553
6	GTR + G	Cox1^2^, Cox2^1^, Cox3^2^, Cytb^3^	4618–6169, 6170–6862, 7726–8521, 12364–13507
7	GTR + G	Atp6^1^, Atp6^2^, Atp6^3^	7049–7734, 7050–7734, 7050–7734, 7051–7734
8	GTR + G	ND6^1^, ND6^2^, ND6^3^	13508–14033, 13509–14033, 13510–14033

Note

Superscript numbers refer to codon position 1, 2, or 3.

## 3 RESULTS

**Figure 3:**
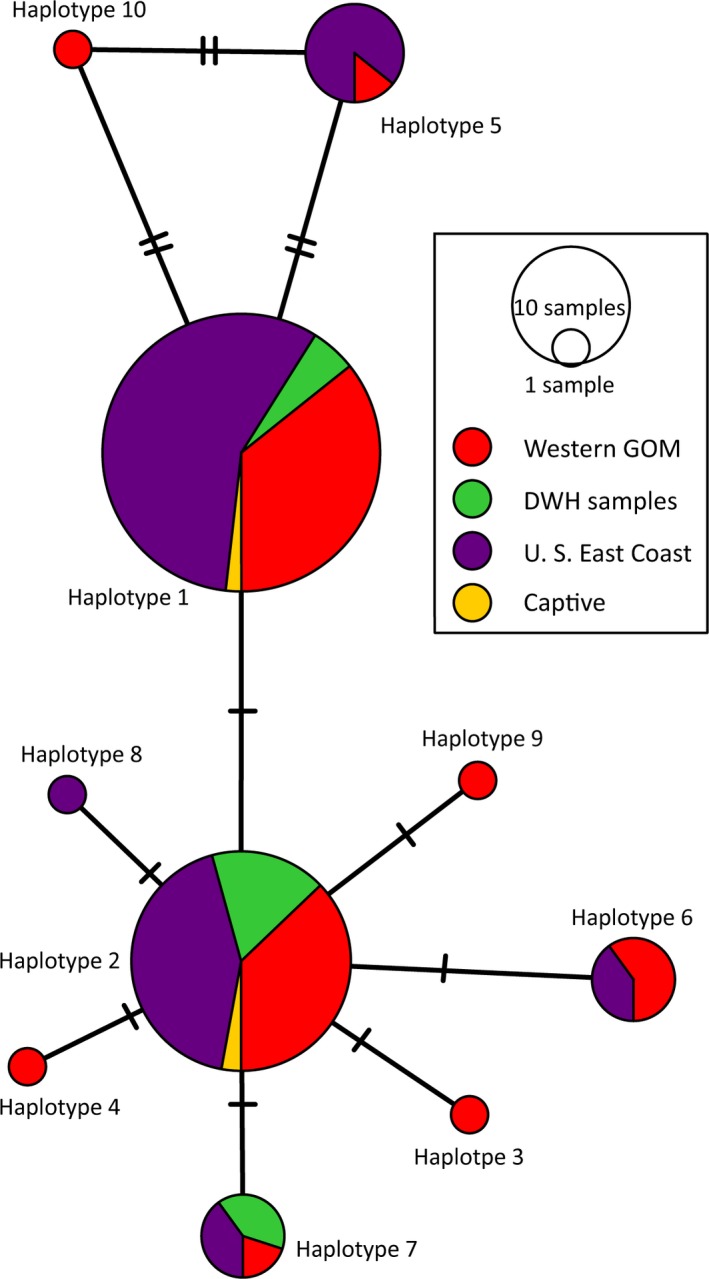
Minimum‐spanning haplotype network of *Lepidochelys kempii* based on mitochondrial control region sequences. Circles represent the ten unique haplotypes found within the samples. Size of the circle is proportional to the number of samples belonging to that haplotype. Colors represent the group designation of the samples: Red—Western Gulf of Mexico, green—DWH samples, purple—U.S. East Coast, and yellow—captive samples. Notch marks on the lines represent mutational steps between haplotypes

**Figure 4:**
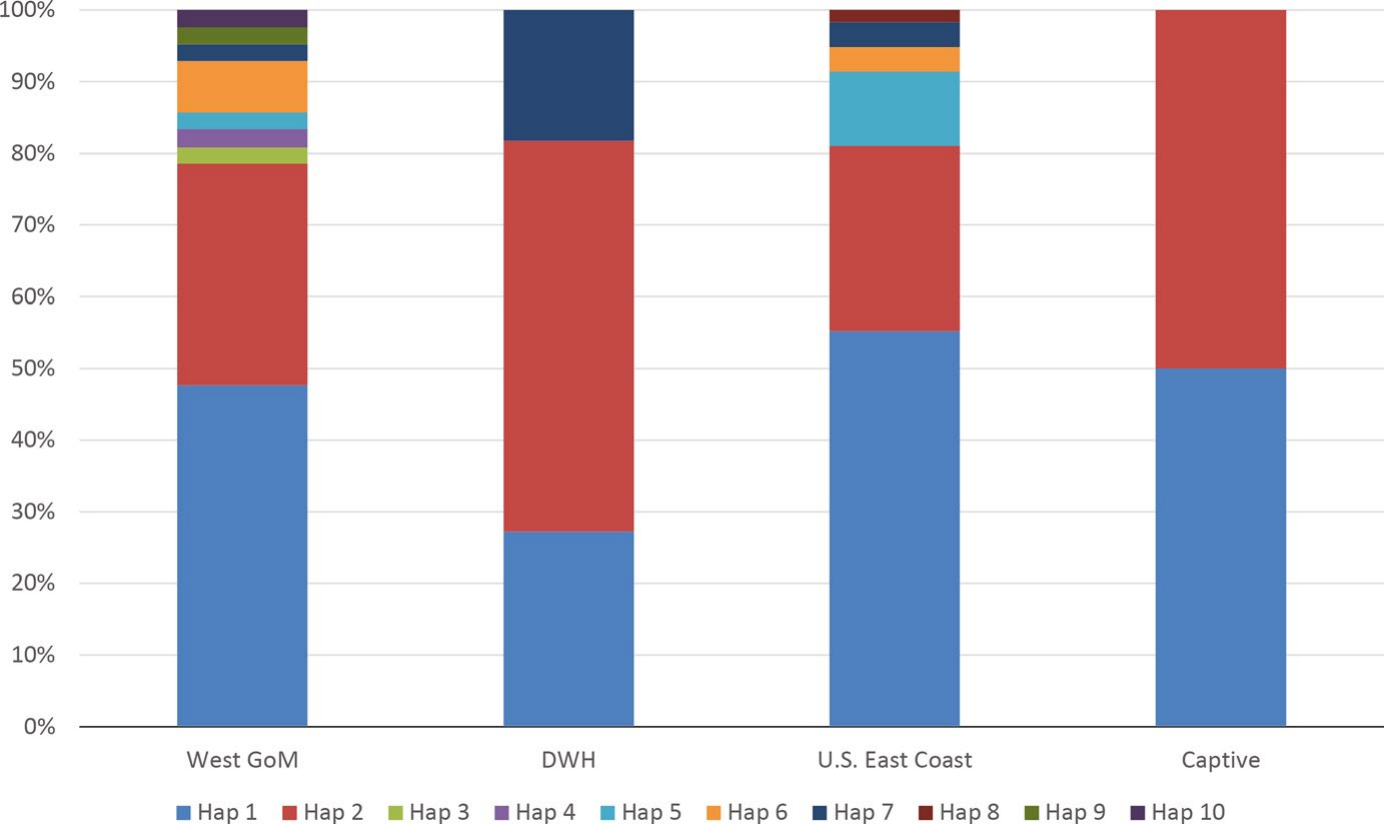
Distribution of the ten *Lepidochelys kempii* haplotypes based on the mitochondrial control region

The control region was sequenced for 113 samples, resulting in ten unique haplotypes within the dataset. Eight of these haplotypes match those identified by Frey et al. (2014) Lk 1.1, 2.1, 3.1, 4.1, 5.1, 6.1, 6.2, and 7.1. Individuals with haplotype 1 (Lk 4.1) are highly abundant, comprising 49.6% of all samples (Figure 3). Haplotype 2 (Lk 6.1) comprises 25.7% of all samples, manifesting strongly in the Western Gulf, U.S. East Coast, and DWH samples (Figure 3). Haplotypes 5 and 10 are more closely related to Haplotype 1, while the remaining Haplotypes 3, 4, 6, 7, 8, and 9 radiate from Haplotype 2 (Figure 3). Approximately 79% of the haplotypes found on the Texas coast are Haplotypes 1 and 2 (Figure 4). Samples taken after the 2010 BP DWH oil spill reflect a similar frequency of 82% but are predominantly Haplotype 2 (Figure 4). Samples collected from the U.S. East Coast have an 80% frequency for Haplotypes 1 and 2 but are predominantly Haplotype 1 (Figure 4). Two previously undefined haplotypes are found as follows: Haplotype 3 from a nesting female sampled in Texas and Haplotype 8 from a sample collected on the U.S. East Coast (Table 4). Pairwise fixation index (ΦST) comparisons between the predefined groups did not yield statistically significant differences (*p* > .05 for all comparisons). Furthermore, the ΦST values are very low (DWH vs. Western Gulf, ΦST = 0.04, *p* = .18 and U.S. East Coast vs. Western Gulf, ΦST = 0.006, *p* = .52). Values close to zero indicate samples are homogenous.

**Table 4:** Number of *Lepidochelys kempii* individuals of each haplotype found in each region, and relation to haplotypes described in Frey et al. (2014)

Haplotype	Frey et al. (2014) Correlation	Western Gulf	DWH	U.S. East Coast	Captive
Haplotype 1	Lk 4.1	20	3	32	1
Haplotype 2	Lk 6.1	13	6	15	1
Haplotype 3		1	0	0	0
Haplotype 4	Lk 5.1	1	0	0	0
Haplotype 5	Lk 3.1	1	0	6	0
Haplotype 6	Lk 2.1	3	0	2	0
Haplotype 7	Lk 1.1	1	2	2	0
Haplotype 8		0	0	1	0
Haplotype 9	Lk 6.2	1	0	0	0
Haplotype 10	Lk 7.1	1	0	0	0

Among the three geographic sampling areas, haplotype diversity (Hd) varied from 0.685 ± 0.0029 (Western Gulf) to 0.626 ± 0.052 (East Coast) (Table 5). Values close to one indicate samples display the maximum diversity of haplotype distribution. Nucleotide diversity (*π*) varied from 0.00134 ± 0.0002 (Western Gulf) to 0.00100 ± 0.00023 (DWH) (Table 5). The Western Gulf samples had significant Tajima's *D* (−1.47505, *p* = .05) and Fu's *F* (−4.386, *p* = .0322) statistics (Table 6). The maximum number of nucleotide differences between any two sequences was 4 (Table 6). For all geographical regions combined, *h* = 10, Hd = 0.658 ± 0.033, *π* = 0.00133 ± 0.00013, and the average number of nucleotide differences between pairwise sequences was 1.013 (Tables 5 and 6).

**Table 5:** Summary statistics of haplotypes found within the three geographical sampling regions

Region	*n*	Prob.	*h*	Hd	Hd *SD*	S	Θ	Θ *SD*	*π*	*π SD*
Western Gulf	42	0.95	9	0.685	0.0029	9	0.00274	0.00117	0.00134	0.0002
DWH	11	0.83	3	0.655	0.111	2	0.00089	0.00068	0.00100	0.00023
East Coast	58	0.97	6	0.626	0.052	6	0.00117	0.00081	0.00132	0.00019
All	111	0.98	10	0.658	0.033	10	0.00248	0.00096	0.00133	0.00013

Note

*n* = number of sequences; Prob. = probability of capturing the deepest coalescent event (*n *− 1)/(*n* + 1); *h* = number of haplotypes; Hd = haplotype diversity; Hd *SD* = +1 standard deviation for Hd; S = number of polymorphic sites; Θ = mutation‐scaled effective population size; Θ *SD* = +1 standard deviation for Θ; *π* = nucleotide diversity; *π SD* = +1 standard deviation for *π*.

**Table 6:** Summary statistics of haplotypes found within the three geographical sampling regions, continued

Region	*n*	*D*_T_	Prob.	*R*_2_	Prob.	Max. *k*	Average *k*	Fu's Fs	Prob.
Western Gulf	42	−1.47505	0.05	0.0566	0.036	4	1.026	−4.386	0.0322
DWH	11	0.36189	0.6732	0.1909	0.3296	2	0.764	0.071	0.5588
East Coast	58	−0.55112	0.3482	0.0839	0.3196	4	1.007	−0.86	0.3994
All	111	−1.17044	0.124	0.0506	0.11	4	1.013	−3.759	0.0924

Note

*n* = number of sequences; D_T_ = Tajima's D statistic; Prob. = significance as determined by coalescent simulation; *R*_2_ = Ramos‐Onsins and Rozas' (2002) *R*_2_ statistic; Max. *k* = maximum number of pairwise nucleotide differences; Average *k* = average number of pairwise nucleotide differences; Fu's Fs = Fu's Fs statistic.

**Figure 5:**
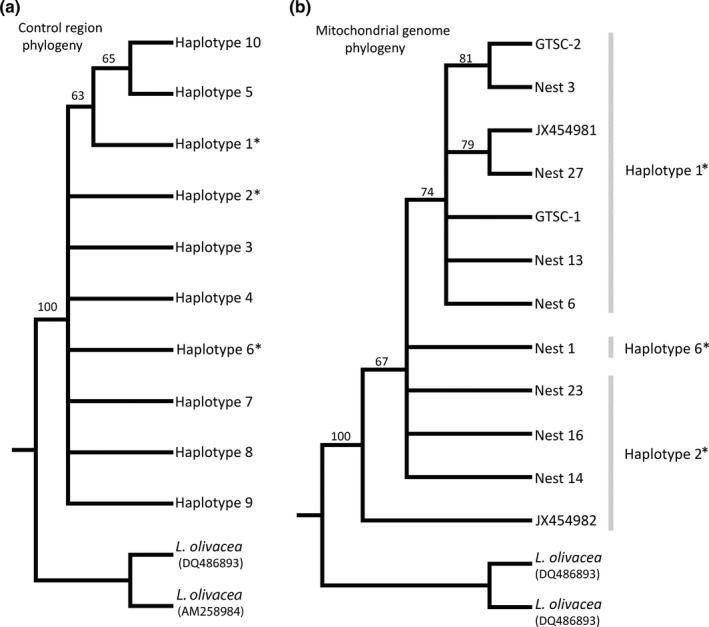
Maximum‐likelihood phylogenetic reconstruction of *Lepidochelys kempii* samples. Numbers on branches indicate bootstrap support. Branches with less than 50 are collapsed. (a) Phylogeny based on mitochondrial control region haplotypes. (b) Phylogeny based on complete mitochondrial genomes. *denotes the three control region haplotypes that were identified within the twelve mitochondrial genomes

The maximum‐likelihood phylogenetic reconstruction based on the ten haplotypes of the control region does not resolve the relationship between these haplotypes, with most branches collapsed due to weak support (Figure 5). Only Haplotypes 1, 5, and 10 are grouped in a strongly supported clade with Haplotype 1 basal to Haplotypes 5 and 10.

**Figure 6:**
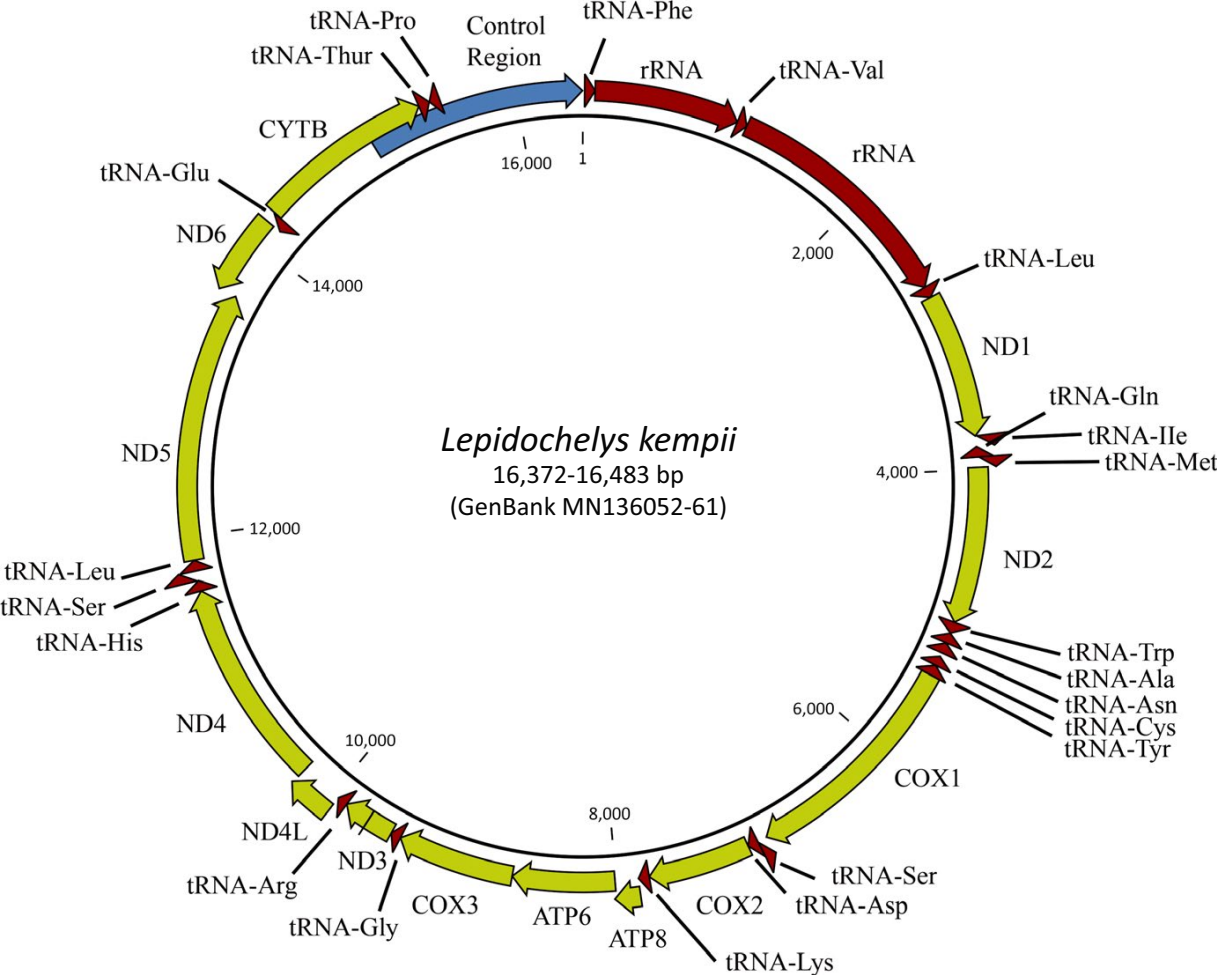
Mitochondrial gene arrangement of *Lepidochelys kempii*

Ten mitochondrial genomes were successfully sequenced and compared to two partial genomes present on GenBank (Accession JX454981, JX454982) (Table 3). The ten genomes range in length between 16,372 and 16,483 bp. The Kemp's ridley mitochondrial genome consists of 12 protein‐coding genes: ND(1‐6,4L), ATP6 and ATP8, COX(1‐3), 22 TRNAs, 2 RNAs, and a large spacer segment containing the control region (Figure 6). Eight out of twelve protein‐coding genes are separated by a tRNA sequence (Figure 6). This is the same gene arrangement in the two partial genomes downloaded from GenBank.

The mitochondrial genome displayed the greatest number of haplotypes (h = 12) and the highest level of haplotypic diversity (Hd = 1) (Table 7). Gene 16s was the longest gene, had the greatest number of haplotypes among all genes (h = 4), and the greatest number of variable sites (*n* = 31), but a lower haplotype diversity (Hd = 0.4545) compared to the control region (Hd = 0.5909) and COX3 (Hd = 0.5909) (Table 7).

**Table 7:** Characteristics of genes within the *Lepidochelys kempii* mitochondrial genome

	*h*	Hd	Number of variable sites	Length (bp)
*Mt* genome	12	1	103	16,389
12s	1	0	0	968
16s	4	0.4545	31	1,616
ATP6	1	0	0	683
ATP8	1	0	0	165
Control region	3	0.5909	2	764
COX1	2	0.1667	17	1,548
COX2	1	0	0	693
COX3	3	0.5909	3	785
CYTB	3	0.3182	13	1,144
ND1	3	0.4394	10	973
ND2	1	0	0	1,042
ND3	1	0	0	350
ND4	2	0.1667	2	1,381
ND4L	1	0	0	299
ND5	2	0.1667	1	1,800
ND6	1	0	0	526

Note

h, number of haplotypes; Hd, haplotype diversity.

The ten mitochondrial genomes recovered match three previously defined haplotypes (based on the control region): Lk 2.1, 4.1, 6.1 (Frey et al., 2014). One sample collected from a nesting female on South Padre Island matches Lk 2.1 (control region Haplotype 6, this study). Four nesting samples, two GTSC samples, and GenBank JX45981 match Lk 4.1 (control region Haplotype 1, this study). Three samples from nesting females, as well as GenBank JX454982, match Lk 6.1 (control region Haplotype 2, this study). The mitochondrial genome sequences are 99.12%–99.90% identical to each other with number of differences in alignment positions ranging from 2 to 144.

The partitioned maximum‐likelihood phylogenetic reconstruction using complete mitochondrial genomes results in a better resolved tree than that based on the control region alone (Figure 5). GenBank JX454982 (control region Haplotype 2) forms the basal branch. This is followed by four unresolved branches (3 belong to control region Haplotype 2 and 1 to control region Haplotype 6). These unresolved branches form a polytomy with a strongly supported clade of specimens belonging to control region Haplotype 1. This control region Haplotype 1 clade is made up of a polytomy of three unresolved branches, and two supported clades (Figure 5).

## 4 DISCUSSION

All but one of the six haplotypes documented by Frey et al. (2014), present in 2003 and 2006, are found in this study. The Western Gulf haplotype frequencies are indicative of the 82% frequency of Haplotypes 1 and 2 previously found in Frey et al. (2014). Two individuals sampled in this study returned a haplotype not previously referenced. Although Frey et al. (2014) do not provide enough data to estimate haplotype diversity for direct comparison with our results, the data that are provided can be used to estimate the maximum diversity possible in their study assuming maximum equitability. Frey et al. report that the combined frequency of the two most common haplotypes is 82% while the remaining 4 make up 18%. Assuming maximum equitability, 41% frequency for each of the two most common haplotypes and 4.5% frequency for each of the four remaining haplotypes would yield a maximum haplotype diversity of 0.66. The haplotype diversity for our population in South Padre Island, Texas, is slightly higher at 0.69. Since the Frey et al. (2014) study spanned 11 years and over 500 samples, this suggests that perhaps new haplotypes are being introduced into the breeding stock of South Padre Island.

A greater number of haplotypes are found on the Western Gulf than on the U.S. East Coast or within the DWH samples (Table 5). This is consistent with the fact that the majority of the population nests in northern Mexico, with a growing number documented along the Texas coast (Shaver & Caillouet, 2015; Shaver, Rubio, et al., 2016). The significant Tajima's D and Fu Fs statistics indicate the Western Gulf population may be undergoing population expansion (Table 6). This is further supported by our evidence of additional haplotypes and higher haplotype diversity compared to earlier studies (Frey et al., 2014). Population analyses of the U.S. East Coast samples are not warranted because they were taken from juveniles and therefore cannot be considered a separate population as these individuals may have originated from nesting beaches in Mexico or Texas (Pritchard & Márquez, 1973; Putman, Mansfield, He, Shaver, & Verley, 2013; Putman, Shay, & Lohmann, 2010).

The turtles sampled after the 2010 BP DWH oil spill are likely transient turtles from the Western Gulf, utilizing developmental, foraging, and migratory habitats (Hart et al., 2018; Reich et al., 2017; Seney & Landry, 2008, 2011; Shaver et al., 2013; Shaver, Hart, et al., 2016; Shaver & Rubio, 2008). The haplotype frequencies of the Western Gulf and the BP DWH oil spill samples are similar. Haplotype 2 is the dominant of the three haplotypes present in the DWH samples, while Haplotypes 1 and 2 are almost equivalently present in the Western Gulf samples. The combined frequency of Haplotypes 1 and 2 in the Western Gulf samples is about 80%, also similar to the reported combined frequency of these two haplotypes in Frey et al. (2014). The population pairwise fixation index test results in an ΦST value close to zero between the DWH samples and Western Gulf samples, demonstrating the two groups could be from the same population as they are genetically very similar and the existing differences are not significant.

The total frequency distributions for Haplotypes 1 and 2 are nearly identical between the Western Gulf and U.S. East Coast samples. The Haplotype 1 and 2 frequencies are indicative of those found from the Western Gulf, DWH samples, and Frey et al. (2014). Additionally, when comparing the U.S. East Coast samples to the Western Gulf samples, the resulting fixation index is also close to zero. This demonstrates that these two groups are also genetically similar and cannot be considered separate populations.

The two captive samples belong to two separate haplotypes. The captive Kemp's ridley from the Western GoM matches Haplotype 1 (Lk 4.1), and the captive Kemp's ridley from the U.S. East Coast matches Haplotype 2 (Lk 6.1). Both these haplotypes are found on either coast; however, the U.S. East Coast samples in this study typically belonged to Haplotype 1 (Lk 4.1).

After complete assembly of ten mitochondrial genomes, it was determined that most of the variation in the Kemp's ridley mitochondrial DNA occurs in the hypervariable control region, evidenced by the number of haplotypes present, haplotypic diversity, and sequence length compared to the other markers (Table 7). Ribosomal RNA 16s contained the greatest number of haplotypes of all markers, but the greatest haplotype diversity was found in the control region (Table 7). Gene COX3 had equivalent haplotype number and haplotype diversity values when compared to the control region, but within a longer sequence. When looking at the complete mitochondrial genome, it is also evident that some genes are more variable than others. Genes ATP6, ATP8, ND2, ND3, ND4L, ND6, and COX2 and the ribosomal RNA 12s are all perfectly conserved across individuals. For COX1 and ND4, only one out of ten individuals shows variation in the gene. The ribosomal RNA 16s varies across multiple individuals, as do the genes ND1, ND5, and CYTB. Gene COX3 is the least conserved of all genes.

Targeting solely the control region limits the detection of variation between individuals. This is apparent from the phylogenetic reconstruction where the tree based on complete mitochondrial genomes shows greater bootstrap support and more resolved branches. Though patterns can be seen within the control region, this study indicates that full genomes convey a more robust analysis. Targeting solely the control region is adequate for assigning individuals into haplotype groups; however, specimens still contain differences from each other within the rest of the mitochondrial genome. Analysis of full mitochondrial genomes results in more haplotypes and provides greater resolution for phylogenetic reconstruction. Though it must also be considered that the poor resolution in the phylogenetic reconstruction based solely in the control region is partly due to the multifurcation shown by the star‐shaped haplotype network which cannot be represented well by bifurcation as required in phylogenetic reconstruction (Posada & Crandall, 2001). Nevertheless, using full mitochondrial genomes do provide greater genetic resolution whether for phylogenetic reconstruction or population level analyses. The twelve mitochondrial genomes showed twelve unique haplotypes. If only the control region is analyzed for these twelve specimens, the number of haplotypes is reduced to only three, showing a dramatic loss of resolution.

Furthermore, haplotypes based on the control region may lead to erroneous conclusions. For example, the mitochondrial genome of Genbank JX454982, which belongs to control region Haplotype 2, is clearly different from the mitochondrial genome of Nests 16, 14, and 23, which also belong to control region Haplotype 2. The phylogenetic analyses based on complete mitochondrial genomes show that the three nest samples actually belong to a clade containing members of control region Haplotypes 6 and 1 (Figure 5), with JX454982 basal to this clade. Past studies have relied on the mitochondrial control region to determine levels of variation between individuals. This study indicates that they may have underestimated diversity within the Kemp's ridley population, but more importantly, they may have missed genetic structuring due to lack of resolution.

Though future studies should undoubtedly target nuclear regions, the use of mitochondrial genomes will remain invaluable because of its maternal inheritance and Kemp's ridley female nesting site fidelity. This study demonstrates that the mitochondrial genome of these turtles can be obtained directly from genomic DNA using next generation sequencing technology without the need of amplification. The ten genomes obtained in this study were multiplexed with eighty‐six other libraries in the Illumina HiSeq 2500. This resulted in genome sequences with an average read depth of more than 100 and a minimum of 30. To put this in perspective, the current standard for calling single nucleotide polymorphisms (SNP) in the human genome is a read depth of 30. This number is based on Bentley et al. (2008) research, where they show that the number of SNPs reaches an asymptote after a read depth of 30. Furthermore, they show that this asymptote is reached even sooner (read depth = 10) for homozygous SNPs. Since the mitochondrial genome is homozygous (single stranded and maternally inherited), then a minimum read depth of 10 would result in high quality sequences. Based on our study, a single lane of Illumina HiSeq 2500 should be able to generate 288 mitochondrial genomes with a minimum read depth of 10 and an average read depth greater than 30. This simplified method presented in this study for obtaining complete mitochondrial genomes and the increased genetic resolution that they provide demonstrate that future genetic studies of the Kemp's ridley should target the entire mitochondrial genome and not solely the control region.

## 5 CONCLUSION

Congruent with previously published research, our results indicate that the control region is the most variable region in the Kemp's ridley mitochondrial genome. We demonstrate a cost‐efficient method of obtaining mitochondrial genomes in sea turtles and show that analysis of Kemp's ridley complete mitochondrial genomes will result in greater genetic resolution than analysis of the control region alone. Future studies with marine turtles should utilize full genomes for a greater understanding of genetic diversity of within populations. We show that the genetic makeup of deceased turtles stranded after the *Deepwater Horizon* oil spill is indistinguishable from the breeding stock in South Padre Island, Texas, indicating that the stranded turtles likely originated from nesting beaches along the Western GoM coast. Our results demonstrate that the genetic diversity of the critically endangered Kemp's ridley, despite large population declines, is stable. Routine genetic analyses of populations, such as ours, provide vital information for managers establishing recovery priorities and developing conservation strategies for critically endangered species.

## CONFLICT OF INTEREST

The authors declare they have no conflicts of interest. Sea Turtle, Inc. is a nonprofit organization focused on sea turtle rehabilitation, conservation, and public education and is not a competing interest.

## AUTHOR CONTRIBUTIONS

DF conceptualized the study; DF, HRF, and JAG designed the methodology; DF, JAG, and HRF acquired funding; DF, JAG, and HRF provided resources and acquired the data; DF and HRF analyzed and interpreted the data; DF and HRF drafted the manuscript; and DF, HRF, and JAG revised and approved the manuscript to be submitted.

### OPEN DATA BADGE

This article has earned an Open Data Badge for making publicly available the digitally‐shareable data necessary to reproduce the reported results. The data is available at https://www.ncbi.nlm.nih.gov/genbank/.

## Data Availability

Mitochondrial genome and haplotype DNA sequences can be accessed online through GenBank.
